# Cytologic studies of the fallopian tube in patients undergoing salpingo-oophorectomy

**DOI:** 10.1186/s12935-016-0354-x

**Published:** 2016-10-01

**Authors:** Hao Chen, Robert Klein, Stacy Arnold, Setsuko Chambers, Wenxin Zheng

**Affiliations:** 1 Department of Pathology, University of Arizona College of Medicine, Tucson, AZ 85724 USA; 2 Department of Obstetrics and Gynecology, University of Arizona College of Medicine, Tucson, AZ 85724 USA; 3 University of Arizona Cancer Center, Tucson, AZ 85724 USA; 4 Department of Pathology, University of Texas Southwestern Medical Center, Dallas, TX 75390 USA; 5 Department of Obstetrics and Gynecology, University of Texas Southwestern Medical Center, Dallas, TX 75390 USA

**Keywords:** Tubal cytology, High-grade serous carcinoma, Serous tubal intraepithelial carcinoma, Early detection, Atypical cytology

## Abstract

**Background:**

Mounting evidence suggests the fallopian tube as the origin for ovarian high grade serous carcinoma (HGSC). We attempted to identify the tubal cytological features that allow us to distinguish malignant from benign conditions.

**Methods:**

Tubal specimens (n = 56) were collected from patients who underwent bilateral salpingo-oophorectomy (BSO) due to various clinical indications. A standard procedure to collect fallopian tube brushings from freshly received surgical specimens was developed. Cytological diagnoses were classified into three categories: benign, atypical, and suspicious for malignancy/malignant. Cytological variables of individual cells and epithelia were subjected to statistical analysis. The fallopian tube histology was used as diagnostic reference for confirmation of cytology diagnosis.

**Results:**

Among the 56 fallopian tube specimens, 2 (3.7 %) showed inadequate cellularity preventing further evaluation, 11 (20.4 %) were diagnosed as malignant or suspicious of malignancy, 7 were atypical, and 36 were benign. The presence of three dimensional clusters (*p* < 0.0001, Fisher’s Exact Test), or prominent nucleoli (*p* = 0.0252, Fisher Exact test) was highly correlated with the diagnosis of malignancy. The suspicious malignant/malignant cytological diagnosis was also highly correlated with presence of HGSC with or without serous tubal intraepithelial carcinoma (STIC).

**Conclusions:**

Tubal cytology may be useful for ovarian cancer screening and early detection.

## Background

The accumulated data of the past decade indicates that the fallopian tube is likely the cellular source of ovarian high-grade serous carcinoma (HGSC), the most common and deadly type of ovarian cancer [1–6]. In addition, our prior study also suggests the fallopian tube as the main organ site of origin for ovarian low-grade serous carcinoma [7]. Increasing attention has focused on elucidating carcinogenic pathways in the tube that might help in diagnosis and management. Gynecologic surgical pathologists have defined the changes within the tubal epithelia including p53 signature [3, 8], secretory cell expansion (SCE) [9], secretory cell outgrowth (SCOUT) [10], and serous tubal intraepithelial lesions including serous tubal intraepithelial carcinoma (STIC) [1, 11–15].

Although it is known that the majority of HGSCs arise within the fallopian tube, particularly in the tubal fimbriated end, ovarian cancer early detection remains difficult and patients still largely present in the clinic at late stages. Considering the proposed tubal origin of HGSC and the observation that non-invasive or early cancers mainly occur within the tubal mucosa, we believe that directly sampling of the fallopian tubal cells may provide a straightforward method for early detection. Study of the exfoliative cytology of fallopian tube epithelial cells has been previously attempted with the admission that it was complex, subject to degenerative changes and variation with menstrual cycle [16, 17]. The aim of these early studies was to recognize fallopian tube cells and to avoid misinterpretation of their presence within the cervical Pap smears. Since then, only two reports have attempted to characterize the tubal cytological features using liquid based cytology. These studies were performed with the aims of minimizing false positive interpretations of cytology specimens obtained in association with risk-reducing salpingo-oophorectomy [18] and development of a screening test for adnexal malignancy [19].

The reasons why tubal cytology studies remain under developed relate mainly to the short timeframe of elucidation of serous carcinogenesis. It is our prediction that tubal cytology may become a practical approach for early detection or clinical decision making prior to prophylactic salpingo-oophorectomy.

With the above understanding, in this preliminary study, we develop a baseline tubal cytology method and study its ability to distinguish malignant from benign conditions affecting the fallopian tube.

## Methods

### Patient selection

An Institutional Review Board approval was obtained from University of Arizona prior to commencing the study. A total of 38 patients were recruited resulting in 56 fallopian tube specimens. In this study, cells from bilateral fallopian tubes were combined into a single vial to generate one specimen for each of 20 patients. The remaining specimens represented samples from a single fallopian tube. Samples from pregnant women were excluded. Patients’ age ranged from 32 to 86 years old with a mean of 55 years, of whom 26 patients were post-menopausal and 12 patients were pre-menopausal. Informed consent was obtained from each patient.

### Fallopian tube epithelium collection

Following surgical excision, a fresh specimen was received and the fallopian tube was identified. Specimens were collected within 30 min after the clamping and placed into the ThinPrep vial. A standard procedure using a cytobrush to collect fallopian tube brushings into a ThinPrep vial was developed. A cytobrush was introduced into the lumen and rotated gently before being withdrawn across the fimbriated surface. The cytobrush containing obtained tubal cells was agitated in 20 ml of Cytolyte, the ThinPrep solution, which contained 10 % formalin to ensure minimal changes of cytology. A Thin Prep, one hematoxylin and eosin (H&E) stained cytospin and two unstained cytospin preparations were made from the cellular sample fluid. The remainder of the fluid was concentrated into a 1 ml vial and frozen at −80 °C.

### Cytology evaluation

All cytology slides were reviewed in a blinded fashion without knowledge of corresponding pathology diagnosis. Three co-authors with cytology expertise individually reviewed the cases and consented diagnostic categories were recorded. Samples were classified into benign, atypical, suspicious for malignancy/malignant categories. A diagnosis of suspicious for malignancy was rendered when less than 2 malignant cell clusters were present in a single sample. The ThinPrep slide was evaluated for cellularity (in quartiles), mesothelial like sheets, tubal epithelium, background (clean, mucin, blood or necrosis), large single cells, three dimensional cluster of cells, nucleoli and anisonucleosis (>4 times) within epithelial cells groups. An arbitrary adequacy criterion of at least five epithelia clusters without apparent mesothelial like sheets in a single specimen was applied.

### Clinicopathologic correlation between cytological diagnosis and permanent pathologic findings

The surgical specimens were processed in the standard manner with the exception that the fallopian tubes were entirely submitted for evaluation. Fisher’s Exact Test was used for correlation between cytological variables and cytological diagnosis. Chi square test was used for the correlation between cytological diagnosis and histological diagnosis. Statistical analyses were performed using JMP 11.2.0 (SAS Institute Inc., Cary, NC).

## Results

### Cytological evaluation of the ThinPrep slide

The cytology slides were reviewed microscopically based on the diagnostic criteria described above. Among the 56 fallopian tube specimens from 38 distinct patients, 2 specimens (from 2 distinct patients) (3.7 %) showed inadequate cellularity (less than 5 benign clusters present) preventing further evaluation, 11 specimens (9 patients) were diagnosed as malignant or suspicious of malignancy, 7 specimens (5 patients) were diagnosed as atypical, and 36 specimens (22 patients) were diagnosed as benign. The age distribution, menopausal status and cytological diagnoses of the patients are summarized in Table 1.

**Table 1:** Distribution of subjects by age, menstrual status and cytologic diagnosis

	Post-menopause		Pre-menopause	
	Age	# Specimens (# patients)^a^	Age	# Specimens (# patients)^a^
Inadequate	74–81	2 (2)		
Benign	54–78	22 (13)	38–55	14 (9)
Atypical	54–69	3 (2)	32–51	4 (3)
Suspicious/malignant	56–86	11 (9)	–	–
Total		38 (26)		18 (12)

^a^A single patient may have two fallopian tube specimens; alternatively both fallopian tubes may have been sampled into a single vial

**Fig. 1:**
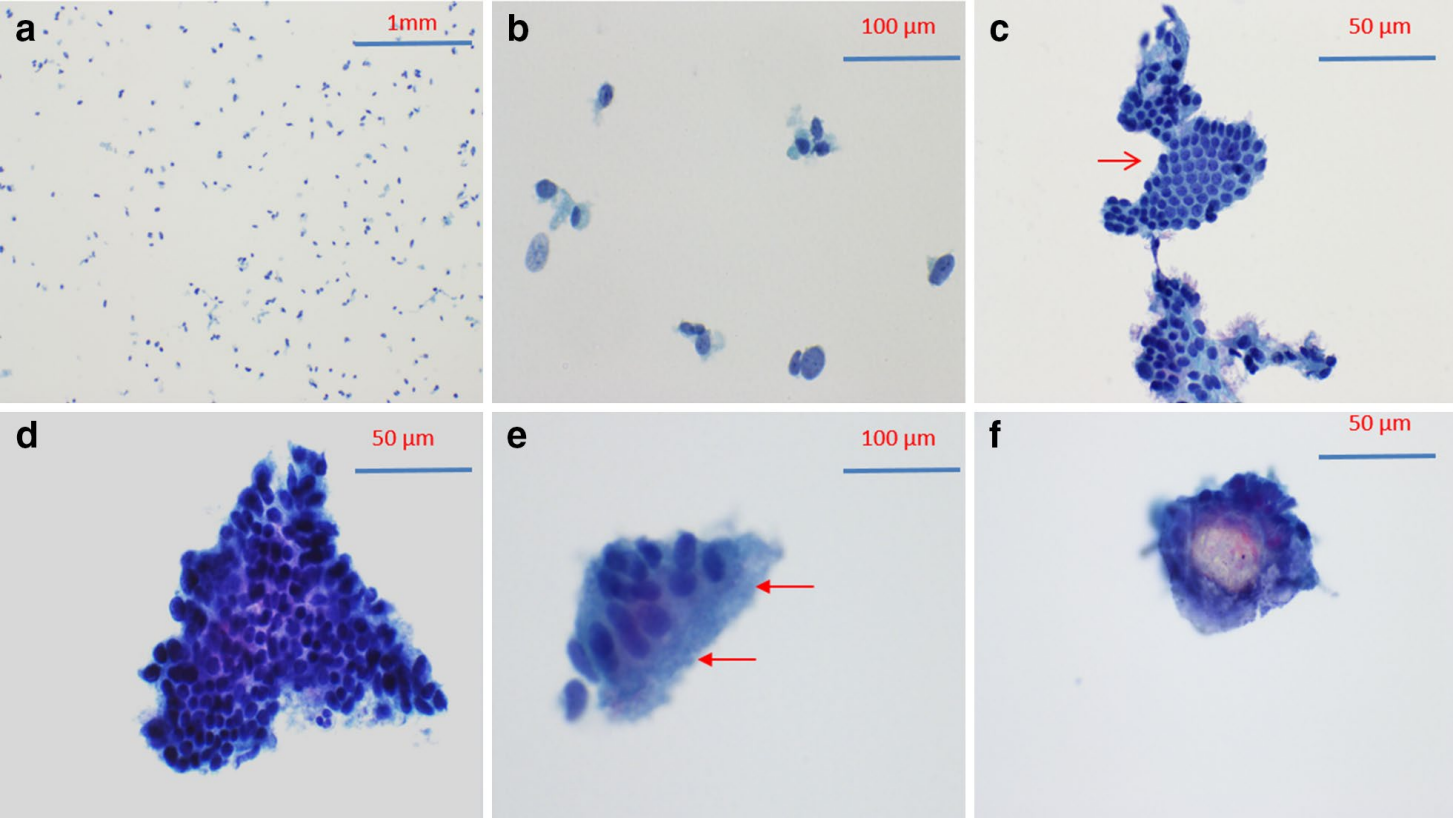
Example of benign tubal specimens. **a** Background cells; **b** Scattered large nuclei most of which are devoid of cytoplasm (bare nuclei); **c** Tubal epithelium with mesothelial-like sheet (*arrow*); **d** Tubal epithelium with angulated sheet containing a mixture of cell types some of which are ciliated; **e** Strip of ciliated epithelium; **f** Psammoma body

Figure 1 illustrates the cytological appearance of benign fallopian tube brushing. The background consists mostly of single cells or small cell cluster (<4 cells). Scattered large cells are present, often devoid of cytoplasm (Fig. 1a). These large cells probably represent tubal secretory cells based on their nuclear characteristics. Benign tubal epithelial clusters are usually angulated sheets containing a mixture of cell types, some of which are ciliated (Fig. 1d, e). Nucleoli are usually inconspicuous. Mesothelial clusters are often seen intermingled with tubal epithelial clusters (Fig. 1c). Psammoma bodies can be occasionally seen within a benign cytologic context.

**Fig. 2:**
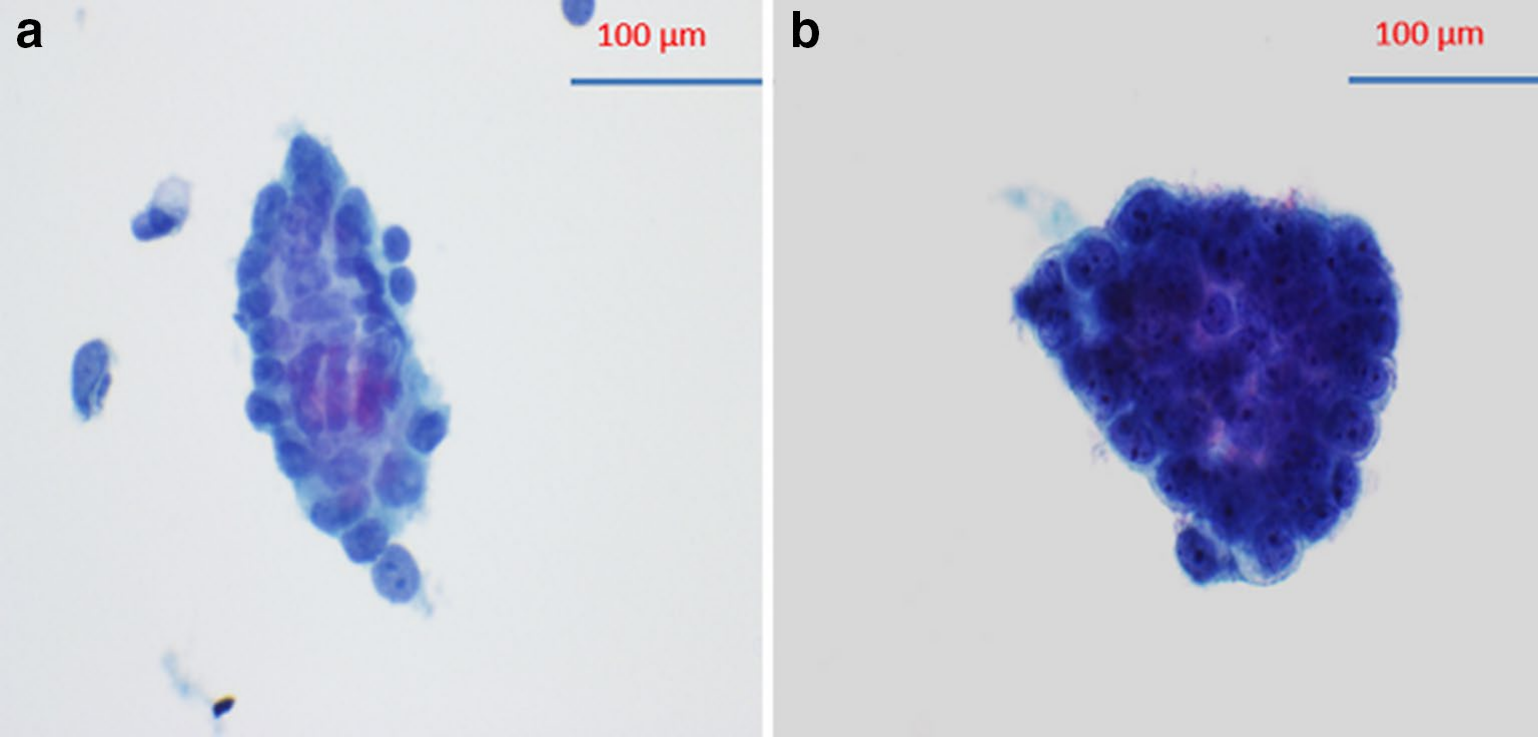
Comparison between atypical cluster without three dimensional clustering (**a**) and malignant cluster with three dimensional cluster (**b**)

Cytological appearance of malignancy shows three-dimensional clusters composed of cells with prominent nucleoli and anisonucleosis (Fig. 2b). Atypical cytology shows angulated sheet composed of atypical cells with small nucleoli and moderate aniosnucleosis. Significantly, three-dimensional clustering is absent (Fig. 2a).

### Correlation between cytological features with cytological diagnoses

At the time of evaluation, individual cases are further evaluated for other cytological features, including anisonucleosis (>4 times) within epithelial cell groups, large cherry red nucleoli, small cherry red nucleoli, mesothelial like sheets, large single cells, and three dimensional clusters of cells. Among all these variables, the presence of three dimensional clusters and large cherry red nucleoli show significant correlation with a malignant cytology diagnosis (see Table 2 for details). The presence of three-dimensional clusters (p < 0.0001, Fisher’s Exact Test), or prominent nucleoli (p = 0.0252, Fisher Exact test) increase the likelihood that the cytologic diagnosis will be suspicious or malignant. In addition, lack of nucleoli decreases the likelihood that the cytologic diagnosis will be suspicious for malignancy (p = 0.0494).

**Table 2:** Correlation of cytological findings with malignant cytological diagnoses

	Aniso nucleosis (±)	Large cherry red nucleoli (±)	Small cherry red nucleoli (±)	Large cells (±)	Mesothelial like sheets (±)	3 dimensional cluster (±)
Malignant	7/4	9/2	2/9	4/7	5/6	11/0
Non-malignant	17/26	1/42	20/23	39/4	21/22	NA
p value	*	0.0252	*	*	*	<0.0001

* p > 0.05

### Correlation between cytological diagnoses with permanent histological diagnoses

**Fig. 3:**
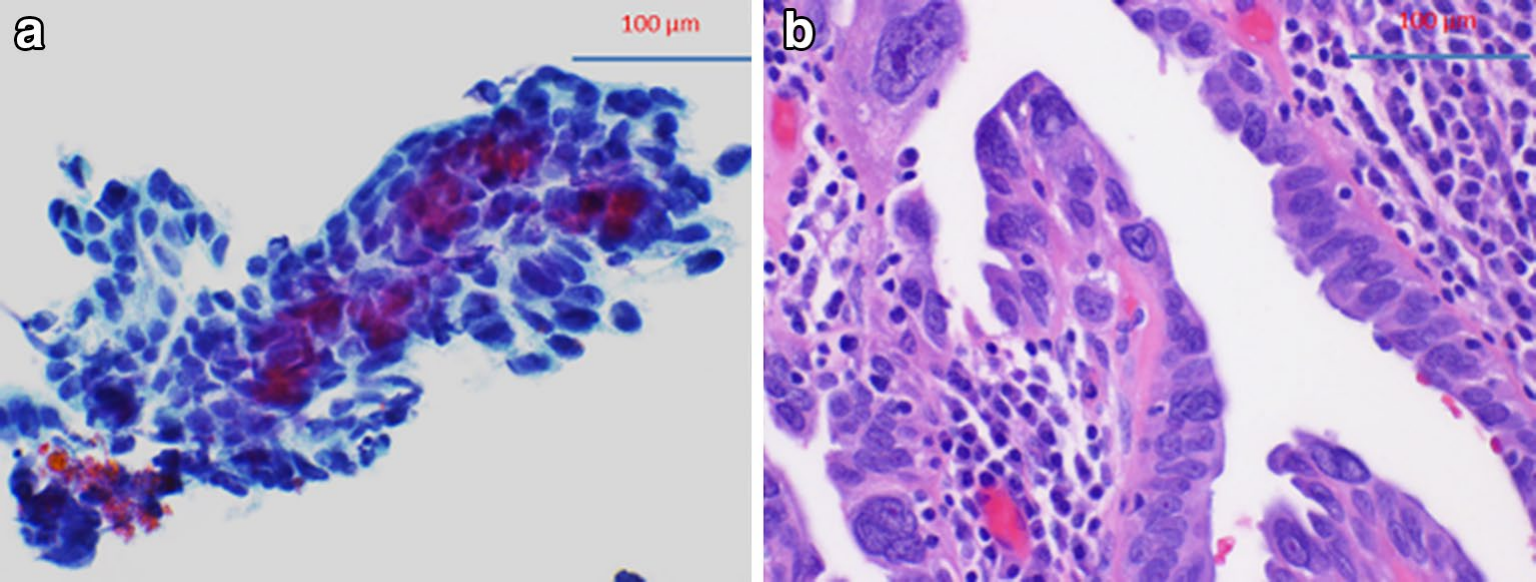
Correlation between cytological and histologic appearance of intratubal neoplasm. **a** Cytological intratubal neoplasia. Cytological intratubal neoplasia showing acquisition of three dimensional contour and significant anisonucleosis and large cherry red nucleoli within the context of angulated sheet of normal epithelial cells. **b** Corresponding surgical pathology section with tubal intraepithelial carcinoma

The cytological diagnosis of malignant or suspicious for malignancy is highly correlated with the histological diagnosis of HGSC (100 %). All malignant cytology cases show ovarian HGSC on histology. Interestingly, two malignant cytology specimens (from two different patients) show no histological fallopian tube involvement. In addition to HGSC, we also identified two cases where concurrent intraepithelial neoplasia was also identified on cytology. In these cases, a HGSC-like cell group is noted within the context of angulated sheet of normal epithelial cells (Fig. 3). We noted that these were the only two cases among all cases involved in the study, which also showed STIC in their permanent histological diagnoses. Therefore, intraepithelial neoplasia on cytological evaluation appears to correlate well with STIC on histological diagnosis. Figure 3 depicts the cytological and histological appearance of such a case.

Atypical cytological diagnoses are usually associated with non-malignant neoplasm or proliferative disorders (including endosalpingosis, paratubal cyst, cystadenoma, borderline tumor, and tubal hyperplasia). However, atypical cytological diagnoses are not significantly correlated with any specific histological diagnoses (data not shown). The correlation of patients’ histological diagnosis with cytological findings is summarized in Table 3. In the 5 cases of atypical fallopian tube cytology, the patient’s age, menopausal status and histological diagnoses are shown in Table 4.

**Table 3:** Correlation of patient’s histological diagnosis with cytological diagnosis

	Benign cytology^a^	Atypical cytology^a^	Malignant cytology^a^
HGSC	–	–	11 (9)
No significant ovarian or fallopian tube pathology	15 (10)	1 (1)	–
Non-malignant/proliferative	21 (12)	7 (5)	–

^a^No. of specimens (No. of patients)

**Table 4:** Age, menopausal status and histological pathology diagnoses in atypical fallopian tube cytology cases

Patient no.	Age	M/PM	Dx
#1 (1 specimen)	47	Pre	Tubal hyperplasia;ovarian serous borderline tumor; endosalpingosis
#2 (2 specimen, bilateral)	51	Pre	Ovarian cystadenoma; peritubal cyst
#3 (2 specimen, bilateral)	69	Post	Ovarian cystadenoma; peritubal cyst
#4 (1 specimen)	32	Pre	Uterine endometrioid carcinoma, peritubal cyst
#5 (1 specimen)	48	pre	No significant ovarian or fallopian tube pathology

## Discussion

By the time of detection, up to 90 % of patients with HGSC are already in the advanced stages of the disease [20]. There is a pressing need for early detection of HGSC. Otsuka and colleagues explored a cytobrush method to examine the samples from endometrial cavity [21]. Although this study showed cytological evaluation can be a useful tool for early detection of pelvic HGSC, the sensitivity of the test from endometrial samples is extremely low. The accumulated data [1–7] in the past decade suggests a tubal origin of ovarian serous cancers. STIC sampling directly from the fallopian tube could be a sensitive way for picking up neoplastic cells when it is not visible grossly. Two previous studies [18, 19] explored the utility of laparoscopic cytologic sampling of benign fallopian tubes and showed promising results. In this study, our main purpose is to characterize the cytological features of malignant and non-malignant fallopian tube epithelium and to test the sensitivity of fallopian tube sampling in detecting HSCG and STIC. Our sensitivity and specificity for identifying HGSC and STIC was 100 %. However, it is to be noted that the cancer cases in this pilot study were all clinically apparent.

The background cells in the thin-prep slide consists of single cells or small clusters (<3 cells). Scattered large cells are present, consisting of 5–20 % of the total background cells. Most of the large cells are devoid of cytoplasm, presenting as large bare nuclei. These large cells are presumed to be secretory cells on the basis of their nuclear size and characteristics. Benign cell clusters usually form angulated cell sheets composed of cells of different size with minimal nuclear atypia. Presence of cilia at the edge of the cluster and intra-cluster mesothelial-like sheets further supports the benign nature of the cluster.

In contrast to the features of benign cytology, among all the cytological variables evaluated, the most prominent cytologic features for malignancy include three-dimensional clusters and large cherry red nucleoli. The benign cell clusters as well as single large cell clusters are frequently seen in the background. Cytological evaluation in this study identified all ovarian HGSC cases (100 %). Two specimens showed no fallopian tube involvement by histology, yet still showed positive result by cytological evaluation. This raises three possibilities: (1) the cytobrush scraped off the lesion site at the time of sampling, (2) since the cytobrushing was done before the tissue specimen was processed for permanent histology, a small or single foci of STIC or tumor may have been completely removed by the cytobrush, or (3) small foci of the lesion remained in the tissue block.

In addition to the benign and malignant groups of tubal cytologic findings, there is a third group of atypical tubal cytology. Although moderate anisonucleosis and even nucleoli can be seen in the atypical group, the atypical cell clusters maintained their angulated contour, compared to the three-dimensional epithelial cell clusters in malignant group. In current study, the atypical cytological diagnoses were associated with a non-malignant proliferative disorder seen via histology. The significance of this correlation is unclear at this stage. Although statistical analysis showed no significant correlation between atypical cytology and any specific non-malignant disorder, this may be due to the limited sample size in the current study. Only 7 specimens (from 5 patients) with atypical cytology were identified. Future studies with a larger sample size may be better able to identify the disorders most associated with an atypical cytology.

Interestingly, cytological evaluation correctly identified both HGSC and intratubal neoplasia in two cancer specimens containing STIC, suggesting the utility of fallopian tube cytology in early detection of the pelvic HGSC. Future studies focusing on developing more sensitive and reliable early detection methods are clearly needed within this area. Alteration of TP53 is known for playing a key role in cancer development [22]. Tubal serous neoplastic lesions including STIC, tubal dysplasia, and even benign looking tubal cells with p53 signature are known to harbor TP53 mutations and stain strongly and diffusely with the p53 antibody [22, 23]. IMP3, an oncoprotein, is a member of insulin-like growth factor II mRNA binding proteins, also known as IGF2BP3 [24, 25]. Overexpression of IMP3 is observed in a series of human malignancies, including ovarian, endometrial, and cervical cancers [26–28]. Our prior study showed that IMP3 may serve as a complimentary biomarker in diagnosing STIC and overexpression of IMP3 in tubal epithelia increases the risk of ovarian serous cancer development [29, 30]. It will be of great interest to examine if tubal cytology in combination with biomarker studies may increase the sensitivity and specificity of detecting serous cancer of tubal origin.

## Conclusions

In summary, this study shows tubal cytology can distinguish malignant from non-malignant and identify precursor lesions such as STIC. In addition, tubal cytology may help to identify non-malignant proliferative disorders. There is potential utility for tubal cytology in screening women at high risk for ovarian or tubal malignancy prior to bilateral risk reducing salpingo-oophorectomy.

## Abbreviations

HGSC: ovarian high grade serous carcinoma
BSO: bilateral salpingo-oophorectomy
SCE: secretory cell expansion
SCOUT: secretory cell outgrowth
STIC: serous tubal intraepithelial carcinoma
